# Eastern monarch larval performance may not be affected by shifts in phenological synchrony with milkweed

**DOI:** 10.1002/ece3.9131

**Published:** 2022-08-04

**Authors:** Sydney M. Gilmour, Heather M. Kharouba

**Affiliations:** ^1^ Department of Biology University of Ottawa Ottawa Ontario Canada

**Keywords:** *Asclepias syriaca*, climate change, *Danaus plexippus*, oviposition preference, phenology, plant–insect interaction

## Abstract

1. Interacting species are experiencing disruptions in the relative timing of their key life‐history events due to climate change. These shifts can sometimes be detrimental to the fitness of the consumer in trophic interactions but not always.
2. The potential consequences of phenological asynchrony for the monarch butterfly (*Danaus plexippus*) and its host plant (*Asclepias* spp.) have not been well‐studied. Given that plants generally undergo seasonal declines in quality, if climate change delays the timing of the larval stage relative to the availability of younger milkweed plants, monarch performance could be negatively affected.
3. Here, we explore the potential consequences for the eastern monarch population due to probable asynchrony with milkweed. We used field surveys around Ottawa, Canada, to determine monarch oviposition preference on common milkweed (*Asclepias syriaca*) plants and the seasonal availability of these plants. To determine the potential fitness consequences when females oviposit on nonpreferred plants, we conducted a field experiment to assess the effect of milkweed size on monarch larval performance (e.g., development time and final size).
4. Preferred oviposition plants (earlier stages of development and better condition) were consistently available in large proportion over the summer season. We also found that declines in leaf quality (more latex and thicker leaves) with plant size did not translate into decreases in larval performance.
5. Our results suggest that even if asynchrony of the monarch–milkweed interaction occurs due to climate change, the larval stage of the eastern monarch may not face negative consequences. Future studies should determine how the relative timing of the interaction will change in the region.

## Graphical abstract

Here we explore the potential consequences for the eastern monarch (Danaus plexippus) population due to probable phenological asynchrony with milkweed, its host plant. We used field surveys around Ottawa, Canada to show that preferred oviposition plants were consistently available in large proportion over the summer season and through a field experiment, we found that declines in leaf quality with plant size did not translate into decreases in larval performance. Our results suggest that even if asynchrony of the monarch‐milkweed interaction occurs due to climate change, the larval stage of the eastern monarch may not face negative consequences.

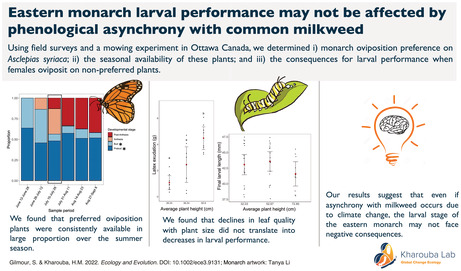

## 1 INTRODUCTION

Climate change is altering species' phenology (i.e., seasonal timing of recurring biological events) at different rates within and across taxonomic groups (Cohen et al., 2018; Thackeray et al., 2016), leading to shifts in the relative timing of key life cycle events of interacting species (i.e., phenological synchrony; e.g., Kharouba et al., 2018; Mayor et al., 2017). Ecological theory predicts these shifts should lead to changes in fitness in pairwise interactions (Cushing, 1969; Hjort, 1914). However, it remains difficult to predict when negative impacts are likely to occur. In trophic interactions, these shifts have led to negative impacts on the fitness of the consumer in some (e.g., Doiron et al., 2015; Plard et al., 2014; Post & Forchhammer, 2007) but not in all contexts (e.g., Reed et al., 2013; Tveraa et al., 2013).

The likelihood of a consumer's fitness being negatively impacted by these shifts depends primarily on two factors (Kharouba & Wolkovich, 2020; Miller‐Rushing et al., 2010; Samplonius et al., 2021): The greater the dependency of a consumer on a single resource and the narrower the seasonal distribution of the resource, the less buffer there is to mitigate the impacts of a disruption in the relative timing of interacting species (e.g., lack of ability to use a different species; Durant et al., 2007; Dunn et al., 2011; Miller‐Rushing et al., 2010). Despite being a critical factor, the length of time the resource is available within a given year is rarely quantified for trophic interactions (Samplonius et al., 2021) and when it has been, the resource is not always seasonally limited relative to the consumer (Halupka et al., 2008; Lany et al., 2016, but see Dunn et al., 2011).

For herbivorous insects, the quality of food available over the season is also an important factor. The quality of many plant species varies seasonally (Mattson, 1980; Schroeder, 1986). Typically, plant nutrient levels (e.g., nitrogen) decrease while physical and chemical defense levels (e.g., cardiac glycosides, tannins) increase over the season (Barton & Koricheva, 2010; Schroeder, 1986). If an insect herbivore emerges too late within a season, its fitness may decrease due to the higher availability of lower‐quality plants (Feeny, 1970). Given seasonal changes in plant quality, choosing high‐quality plants for laying eggs (i.e., oviposition) is important for herbivorous offspring fitness, especially given that young larvae are incapable of traveling to new hosts (Fisher et al., 2020; Futuyma et al., 1984).

Shifts in phenological synchrony between the eastern population of the North American monarch butterfly (*Danaus plexippus* L.) and its host plants (*Asclepias* spp.) due to climate change are likely. As a migratory species, the timing of the arrival and departure of eastern monarchs to and from breeding grounds in the United States and Canada (Cockrell et al., 1993; Urquhart & Urquhart, 1976) is likely to shift due to climate change (Zipkin et al., 2012). Migratory species must adjust the timing of their arrival from often distant grounds—increasing the potential for disruptions in consumer‐resource phenological synchrony (Chmura et al., 2019). Recent evidence based on long‐term monitoring indicates that the timing of the fall migration of eastern monarchs has gotten later over recent decades (Culbertson et al., 2021). There is also some evidence from citizen science data that the timing of arrival of eastern monarchs has gotten later over the past two decades (Howard & Davis, 2015); however, population declines could be an explanation for this change (Brower et al., 2012; Vidal & Rendón‐Salinas, 2014).

Phenological shifts in the monarch's main host plant (common milkweed [*Asclepias syriaca* L.]) have not been well‐studied. To our knowledge, there have been no studies about shifts in the vegetative phenology of common milkweed. However, as the timing of flowering of common milkweed is sensitive to temperature (3.93 days/°C; Howard 2018), and thus also likely the timing of vegetative phenology, the timing of vegetative phenology is likely to change with climate change. Therefore, shifts in the timing of the monarch–milkweed interaction could arise from phenological shifts from either species.

The fitness consequences for the monarch from this type of potential disruption are difficult to predict. There could be negative impacts on fitness given that throughout their range, monarchs use milkweed plants (*Asclepias* spp.) exclusively to oviposit on as adults and feed on as larvae (Ackery & Vane‐Wright, 1984). Alternatively, given some of their life history strategies, they may be insensitive to shifts in synchrony. First, they are thought to have 2–3 generations in our study region (Cockrell et al., 1993; Malcolm et al., 1993). Multivoltine species are hypothesized to be more resilient to shifts in phenological synchrony given the potential for recovery of population size and genetic diversity (Knell & Thackeray, 2016). Second, spring migration to southern Canada is not a single event and occurs as a gradual process (Solensky, 2004). Third, monarchs continue to reproduce (i.e., are not in reproductive diapause) during their spring migration (Urquhart & Urquhart, 1976). These latter two factors suggest that the timing of arrival, and thus oviposition, is not a precise event. Given these life‐history strategies, it is likely that suitable milkweed is available over this time period. However, experimental evidence from the western monarch population shows that larval performance can be constrained by seasonal changes in host plant quality (Yang et al., 2020; Yang & Cenzer, 2020). Studies from the eastern population also show a preference for younger plant tissues or regenerating stems (e.g., Bergström et al., 1994; Haan & Landis, 2019). These findings suggest that there could be a limited window where plants of high quality are available with potential consequences for the monarch.

Here we explore the potential for negative fitness consequences for the eastern monarch population at its northern range limit due to potential shifts in phenological synchrony with common milkweed (*A. syriaca*), the most important host plant species in the summer breeding range (Malcolm et al., 1993; Pocius et al., 2018). We take a first step and consider a simplified hypothetical scenario that shifts in phenological synchrony are driven largely by delays in the timing of arrival of monarchs and/or resulting delays in the oviposition of subsequent generations. We first conduct field surveys around Ottawa, ON, Canada to determine which plant‐level characteristics best predict monarch oviposition preference and to describe the seasonal availability of these preferred plants. If the timing of oviposition is delayed, but preferred plants are available throughout the season, then offspring fitness is less likely to be affected. Based on the results from recent studies on the oviposition preference of the eastern population at the plant level (Fischer et al., 2015; He & Agrawal, 2020), we predict that monarchs will preferentially oviposit on younger milkweed plants.

To explore the potential consequences on larval performance in a scenario where preferred plants are not available through the season, we use a field experiment to test mother how sensitive larval performance is to changes in plant size. If oviposition does not occur on preferred plants, we predict a decline in larval performance (i.e., the preference‐performance hypothesis; Levins & MacArthur, 1969). Based on findings that milkweed leaf quality declines as plants develop (Agrawal & Konno, 2009; Yang et al., 2020; Yang & Cenzer, 2020), we predict that larvae will have higher performance on smaller plants. We assume here that host plant quality is more important than its quantity. We also measure how key milkweed defensive traits differed across plant size. Determining how sensitive larval performance is to plant size will give us a better sense of potential outcomes for the eastern monarch population due to climate change.

## 2 MATERIALS AND METHODS

### 2.1 Study system

The eastern monarch population has declined by more than 80% over the past two decades (Brower et al., 2012; Semmens et al., 2016). As a result, monarchs in Canada are considered *Endangered* by COSEWIC (2016) and listed as *Special Concern* under the Species at Risk Act. The eastern population has a long‐distance autumn migration that is completed in a single generation from breeding grounds as far north as southern Canada to overwintering grounds in central Mexico. The following spring, individuals return to their breeding grounds in the northern United States and southern Canada in 3–4 generations (Solensky, 2004). Once in southern Canada, they produce 2–3 generations (Cockrell et al., 1993; Malcolm et al., 1993). Eggs hatch 3–4 days after they are laid, and then, the larval period typically lasts 10 to 21 days and includes five instars (Oberhauser, 2004).

*A. syriaca* is a flowering perennial herbaceous plant that is native to eastern North America (Fernald, 1950). It can be found along roadsides, and within open grasslands and croplands (Bhowmik & Bandeen, 1976). It reproduces by seed and asexually through its rhizomes (Bhowmik & Bandeen, 1976). In eastern Canada, shoots emerge in spring from April to May and continue to grow until mid‐August to mid‐September when they begin to senesce (Bhowmik & Bandeen, 1976). Their flowering stage is between late June and early August (Bhowmik & Bandeen, 1976). Milkweed plants contain a number of physical (e.g., trichomes, leaf toughness) and chemical (e.g., cyanides and alkaloids) defensive traits (Agrawal & Fishbein, 2006). The stems and leaves contain latex that can be toxic to phytophagous insects. It is made up of high concentrations of cardiac glycosides (also referred to as cardenolides) and cysteine proteases (Agrawal & Fishbein, 2006).

### 2.2 Observational study

#### 2.2.1 Field surveys

**FIGURE 1:**
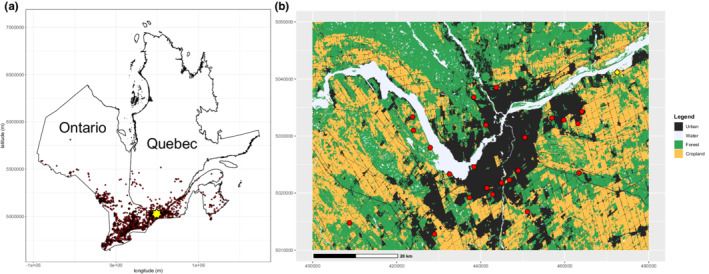
Location of study area (yellow star) relative to northern range limit of the eastern population of the monarch (a) and study sites around Ottawa, ON, Canada (b). Shown in panel a, are monarch sightings in Eastern Canada from citizen science data from eButterfly and Mission Monarch from 2016 to 2018. Shown in panel b are the survey sites (*n* = 24; red dots) and the experimental site (*n* = 1; 45°31′19.3368″ N, 75°20′50.4636″ W; yellow diamond) relative to major land use types (Agriculture and Agri‐Food Canada 2010; https://open.canada.ca/data/en/dataset/9e1efe92‐e5a3‐4f70‐b313‐68fb1283eadf [accessed 15 November 2018]).

We surveyed common milkweed plants for monarch eggs at 24 sites around Ottawa, Ontario, Canada, from June 12 to September 8, 2019 (Figure 1). This period represented the majority of the monarch breeding season according to the range of adult sightings from Journey North citizen science data for the same year (May 27–October 10; https://journeynorth.org/sightings/). Each site was visited six times (i.e., every 12–14 days) within the season. We chose sites that were well distributed across the area, covering a 23 km north–south and a 70 km east–west axis (Figure 1). Sites were old‐field habitats with no current human intervention. Sites differed in their surrounding landscape cover of urban, agriculture, and forest (Figure 1). In the study region, other milkweed species are extremely rare (Kharouba et al. unpubl. data).

To survey milkweed plants, we placed a 1 × 1 m^2^ quadrat at five random locations (chosen for each visit) within a predetermined 100 m transect. Within each quadrat, we made observations on the presence and absence of monarch eggs and larvae on each milkweed stem (i.e., any stem that was separated from another stem by soil). In many cases, these may not be independent plants given the complex, rhizomatous, root system of milkweed plants. However, for simplicity, we use the term “plant” throughout the paper. We also measured the size, vegetative and flowering developmental stages, and herbivory damage on each plant. Following similar criteria and categorical levels used by Fischer et al. (2015), we measured height (from the root crown to the apical leaf), number of leaves, developmental stage of flowers (prebud, bud, anthesis, postanthesis), herbivory (estimated as a percentage of leaf area missing based on four levels: 0%, <5%, 5%–25%, >25%), and leaf discolouration (estimated as the percentage of yellowing leaf area based on four levels: <5%, 5%–40%, 41%–80%, 81%–100%). These characteristics have been found to affect monarch oviposition (e.g., Cohen & Brower, 1982; Fischer et al., 2015; He & Agrawal, 2020; Knight et al., 2019) or offspring performance (Yang et al., 2020).

To supplement these patch‐level egg surveys, we performed broader visual surveys for eggs at each site. For these surveys, two observers began at a random location within the 100 m predetermined transect and walked for 5 min at a constant speed along the transect, checking every milkweed plant within 1 m to their side for monarch eggs and larvae. If a monarch was found, we paused the timer and recorded its location on the milkweed and the milkweed's characteristics. We then resumed the survey until we reached a total of 5 min.

#### 2.2.2 Statistical analyses

To determine the milkweed characteristics that best predict monarch oviposition preference, we used a binomial generalized linear mixed‐effects model (package “lme4”) (Bates et al., 2015). Site was included as a random effect in all models to account for multiple visits and quadrats at the same site. An optimizer (“bobyqa”) was used to improve model convergence. In separate models, we modeled monarch egg occurrence (presence/absence) on milkweed plants from both quadrat and visual surveys as a function of: day of year, plant height (cm), level of leaf discolouration (ordered factor), level of herbivory (ordered factor), and number of leaves. Continuous variables were scaled to improve model convergence. Nonlinearity of day of year, number of leaves, and height were assessed. The model would not converge with the developmental stage (ordered factor), likely because there were too few egg observations in two of the levels (anthesis, postanthesis), so for this factor, we modeled the occurrence of egg and young larvae (i.e., first and second instar larvae). We also considered the impact of milkweed abundance on egg occurrence.

**FIGURE 2:**
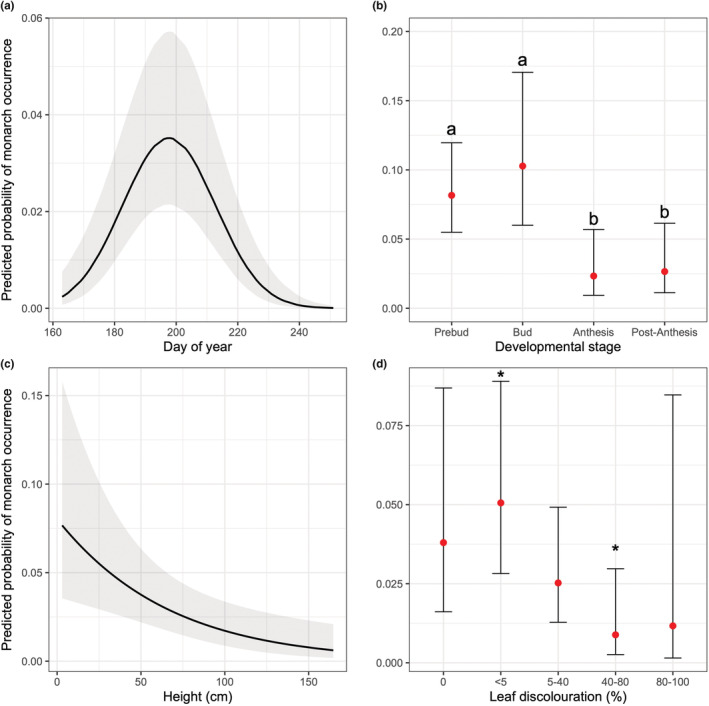
Common milkweed characteristics surveyed that best predict the occurrence of monarch eggs and larvae at 24 sites in Ottawa, Ontario, Canada. Predicted probability of occurrence on milkweed plants based on (a) day of year, (b) developmental stage, (c) height (cm), and (d) level of leaf discolouration (%). In panel b, probability of occurrence includes egg and larval observations. In panels a and c, the line of best fit with 95% confidence intervals based on the conditional effects of the model is shown. In panels b and d, the predicted mean values (red dots) with 95% confidence intervals based on the conditional effects of the model are shown. Models in panels b‐d include day of year and its quadratic term (Table 1). Letters and stars represent significant pairwise comparisons (*p* < .05; Table S3). Raw data in panels a and c are not shown to improve visual interpretation of figures.

Given that there was a mix of predictor classes, we assessed nonindependence between predictor variables using a combination of models depending on the type of response variable (Table S1). Given that the majority of predictor variables showed nonindependence (Table S1), we tested the influence of each predictor on monarch occurrence separately. To account for the seasonal patterns in monarch egg occurrence, the linear and quadratic terms of day of year were also included in each model (monarch occurrence was better predicted by a quadratic relationship; Table 1; Figure 2). We compared the fit of seasonal models (day of year, linear and quadratic terms) to a model that also included a milkweed‐specific variable. To select the best model, we used the difference in the second‐order Akaike information criterion (AICc; >2 ΔAICc) and a χ^2^‐test. We also assessed the overall fit of each model using conditional *R* ^2^ (“r.squaredGLMM” function). Post hoc comparisons between levels of categorical variables were conducted using the “emmeans” function with a Tukey adjustment in the package “emmeans” (Lenth et al., 2018). All statistical analyses were performed using R 3.3–1 (R Core Team, 2018).

**TABLE 1:** The results of model selection amongst characteristics hypothesized to predict monarch occurrence (presence and absence) on milkweed plants (*n* = 24 sites). Models were selected between a reference model (i.e., day of year and day of year^2^) and a model that also included a milkweed‐specific variable (leaf discolouration (%), herbivory (%), developmental stage, height (cm), leaf number, plant abundance) based on ΔAICc. Models with substantial support (>2 ΔAICc) are bold‐faced. Monarch occurrence for developmental stage included eggs and larvae, whereas all other models were based solely on eggs

Reference model	Models	Estimate (SE)	AICc	ΔAICc	χ2‐test	*p* value (df)	*R* ^2^
Day of year + Day of year^2^ (*n* = 2841 plants)			545.0	0			
	**+ Height (cm)**	**−1.61 (0.54)**	**538.2**	**6.82**	**8.82**	**.003 (1)**	**0.52**
	**+ Leaf discolouration (%)**	**N/A**	**539.3**	**5.71**	**13.71**	**.01 (4)**	**0.51**
	+ Herbivory (%)	N/A	545.7	−0.68	5.32	.15 (3)	0.51
	+ Leaf number	2.28 (1.68)	545.4	−0.37	1.62	.2 (1)	0.5
Day of year + Day of year^2^ (*n* = 2830 plants)			741.9	0			
	**+ Developmental stage**	**N/A**	**727.5**	**14.4**	**20.4**	**<.0001 (3)**	**0.46**
Day of year + Day of year^2^ (*n* = 2746 plants)			143.37	0			
	+ Plant abundance	−1.82 (2.81)	144.83	−1.47	0.54	.46 (1)	0.49

### 2.3 Field experiments

#### 2.3.1 Experimental overview

To assess the response of monarch larval performance to plants of different sizes, we conducted a field experiment in an old‐field habitat at the MacSkimming Outdoor Education Centre, Cumberland, ON, Canada (Figure 1). At this site, milkweed is naturally occurring and found in high densities (e.g., 299 stems within a 6 × 6 m^2^ area). The area was last mowed in 2011 for hay but has been left undisturbed since then.

To obtain plants of different sizes, in early summer 2018, we mowed two patches (patch = 5 × 5 m^2^) in total: one patch on June 18 and one on June 25. In early summer 2019, we mowed three patches (patch = 6 × 6 m^2^) in total: one patch on each of June 25, July 2, and July 9. We used a gas weed trimmer machine (STIHL‐FS131R). This resulted in two milkweed size treatment levels in 2018 (small (mowed last) and medium (mowed first)) and three milkweed size treatment levels in 2019 (small (mowed last), medium, and big (mowed first); Figure S1). While the experiments in each year did not have true replication, the initial milkweed height of the different treatment levels (Figure S1) and the environmental conditions experienced by the larvae (mean daily maximum temperature: 26.9°C (2.7 SD) (August 2018) vs. 26.1° C (2.5 SD) (August 2019); https://climate.weather.gc.ca/historical_data/) were similar between years (e.g., medium in 2018 vs. medium in 2019). While mowing has been shown to cause other aspects of plant quality to change (e.g., cardenolides) in addition to plant size in the few days immediately after mowing (Malcolm & Zalucki, 1996), it has been shown not to have detrimental impacts on larval survival (Haan & Landis, 2019). Also, since common milkweed is perennial and can grow vegetatively, we cannot disentangle the effects of size from true age in this experiment.

**FIGURE 3:**
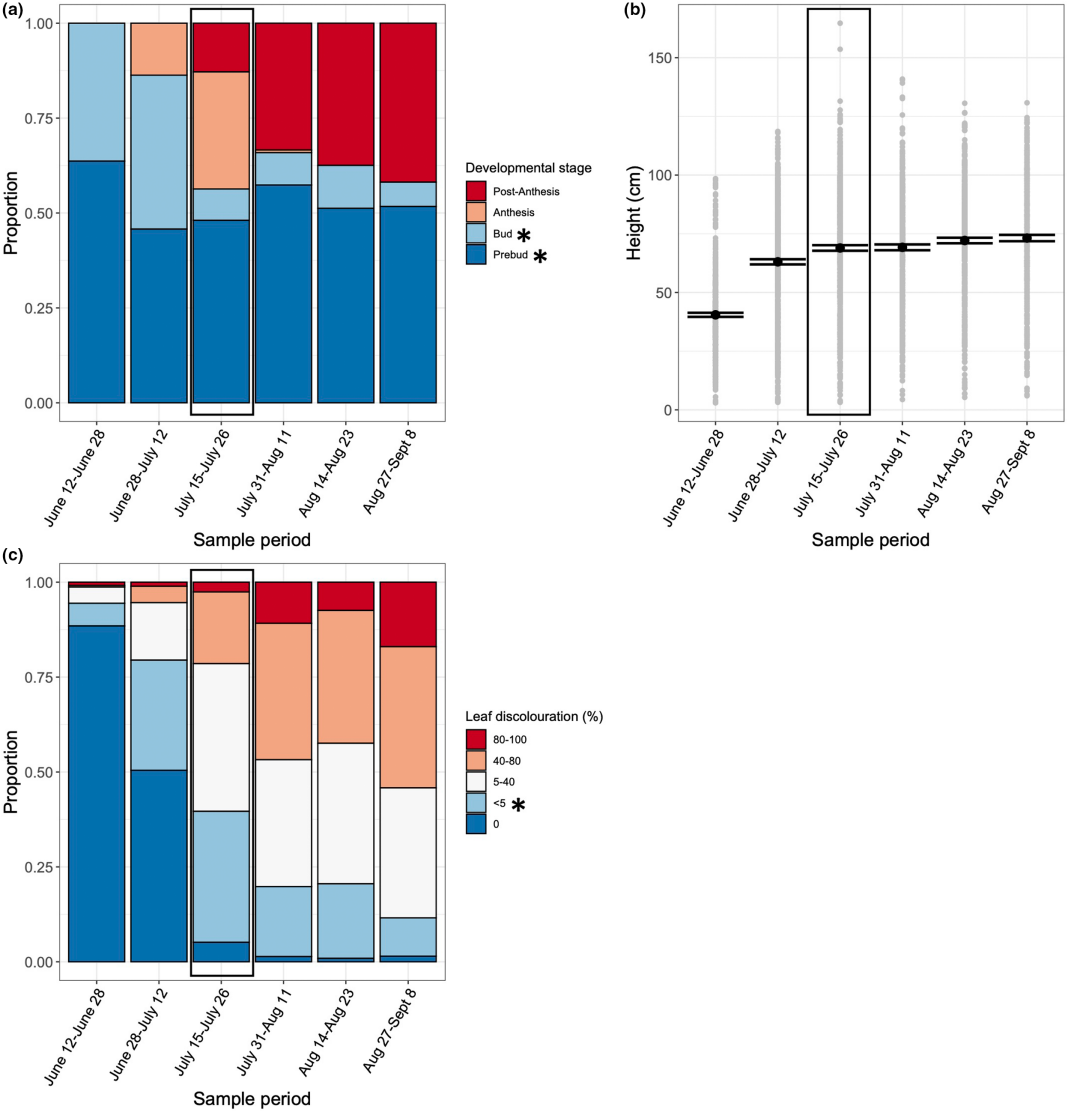
Seasonal patterns of milkweed characteristics based on two‐week sampling periods (*n* = 2851 plants). Shown is the proportion of plants in each level of (a) development stage and (c) leaf discolouration (%). Panel (b) shows all measurements (colored dots) along with the mean value (black dot) of plants in each level of height (cm). Error bars are based on standard error. Black box in each panel represents the timing of the peak egg occurrence. In panels a and c, preferred plant characteristics are noted with a star.

To ensure there were differences in plant size across treatments but minimal differences in environmental conditions experienced by the larvae, we chose a one‐week interval between treatments, which is in line with another milkweed mowing study (Knight et al., 2019). Initial plant height was significantly different across treatments (2018: *F*_1,11_ = 102.33, *p* < .00001; 2019: *F*_2,42_ = 430.39, *p* < .00001; Figure S1). Treatment levels reflected the lower half of the height distribution of the milkweed found across our observational sites surveyed the following day in 2019 (*n* = 7; Figure S1). In 2019, the medium‐size treatment (52.7 cm ± 3.5 SE) was approximately the same as the mean height of plants (57 cm ± 1.7 SE) during the peak egg occurrence (Figures S1 and S2; Figure 3).

#### 2.3.2 Monarch rearing

Monarch eggs came from wild adults that were locally captured for both experiments. Within an experiment, all eggs were introduced to all plants on the same date. In July 2018, we collected monarch eggs and larvae from one site (Vanessa Honey Equestrian Centre, 45°25′36.6564″ N, 75°58′25.5396″ W) in the Ottawa region and kept them in a growth chamber (Biochambers model LTCB‐19) on a 21°C/25°C 15:9 L:D cycle with a peak in light intensity at 12:00 pm. These conditions were chosen to represent the average environmental conditions in July for this region. We fed the larvae fresh milkweed leaves every 1–2 days until they reached pupation. On August 3, 2019, we collected four wild adult monarch butterflies (female *n* = 2, male *n* = 2) from two sites in the Ottawa region (45°22′01.1784″ N, 75°39′06.9588″ W, 45°28′55.3″ N, 75°47′20.5″ W).

Upon pupation (2018) and capture (2019), we placed each adult pair (2018: 1 pair, 2019: 2 pairs) in an enclosure (90 × 60 × 60 Pop‐up Cage, Watkins & Doncaster) in a courtyard at the University of Ottawa, Ottawa, Canada (45°25′16.3884″ N, 75°40′52.644″ W). In each enclosure, we placed two potted hybrid coneflowers (*Echinacea purpurea* “PowWow White” and *Echinacea purpurea* “PowWow Wild Berry”) and two potted milkweed plants (*A. syriaca* and *A. tuberosa*). Mated females oviposited on both milkweed species on August 9, 2019, and within 24 hours of oviposition, eggs were transferred to the experimental site.

#### 2.3.3 Experimental details

On July 30, 2018, we chose 15 random plants from each experimental patch and measured their individual heights and number of leaves. On each marked milkweed plant (*n* = 30), we placed an egg on the underside of two leaves. If both eggs on a given plant hatched (this only occurred three times), within 1–3 days of hatching, we randomly chose one larva to remain on the plant, and the other was placed on a plant without a larva within the same treatment. We used polyethylene insect rearing bags (71 x 48 cm and 100 x 66 cm, Bug Dorm) to cover the plant and secured them with 2–3 metal tent pegs per bag.

On August 10, 2019, we chose 20 random plants from each experimental patch and measured their individual heights and number of leaves. On each identified plant (*n* = 60), one egg was placed on the underside of a leaf from the second upper whorl using the latex from the milkweed as an adhesive. We evenly divided the eggs from the two adult monarch pairs amongst treatment levels. Each plant was then enclosed with a sandbag‐style exclusion bag (120 × 70 cm) (Thomson et al., 2011). These bags were sewn from polyester fabric (~300 μm aperture) and the bottom part of the bag was filled with sand (Quikrete Play Sand).

In both years, every 1–3 days, we took measurements of larval stage and length. We used three estimates of larval performance: development time (days from larval emergence to pupation), final length (mm, defined by the last larval length measurement taken before pupation), and survival from larval emergence until pupation. In the western monarch population, larval length is highly correlated with larval weight (mass = 0.0223 × length^2.9816^, *R* ^2^ = 0.97; Yang & Cenzer, 2020) and larval weight is generally strongly correlated with fecundity in insects (Honēk, 1993 but see Leather, 1988).

Egg hatch failure within treatment levels was 27% in 2018 and varied between 10 and 35% across treatment levels in 2019. Survivorship from larval emergence to pupation varied from 45 to 73% across treatment levels in 2018 and 77 to 94% in 2019. As expected, these values are high compared to that of the survival rates of naturally occurring monarchs (e.g., 12% from egg to pupation; Borkin, 1982) but comparable to experiments that confined larvae with mesh bags as we did here (72% survival rate; Zalucki et al., 2001).

#### 2.3.4 Leaf quality

To determine the influence of plant size on leaf quality, we measured leaf thickness and latex exudation following methods from Agrawal and Fishbein (2006). Both leaf thickness (Agrawal & Fishbein, 2006) and latex exudation (e.g., Malcolm & Zalucki, 1996; Van Zandt & Agrawal, 2004) have been found to affect monarch larval performance. We randomly selected eight A. syriaca plants without monarchs from each treatment level on September 13, 2019. For each plant, we measured their height and recorded leaf thickness (mm) using a digital micrometer (Accusize, Model # MD71‐0001). It was measured as the average of two readings from the first and second lateral veins of a single leaf on the third whorl of every plant. We measured latex exudation by cutting 5 mm off the tip of a leaf and collecting the quantity of exuding latex on 42.5 mm preweighed filter paper. Once all latex was collected on a preweighed disc of filter paper, we placed it on top of another dry preweighed filter paper disc. These discs were subsequently dried at 60°C for 72 h and then weighed again using a microbalance (Sartorius Research R 160 P Electronic Semi‐Microbalance).

#### 2.3.5 Statistical analyses

To determine whether milkweed size influences larval performance, we ran 4 separate models for each year (8 models total) with the following response variables: development time (days), final length (mm), absolute growth rate, and survival (excluding egg hatch failure). We used the mean initial plant height on the first day of the experiment as a measure of plant size as height measurements are quicker to do and are less prone to measurement error than other proxies of size (e.g., leaf number or leaf area). As preliminary analyses suggested an effect of year (environmental conditions and the rearing methods were similar but not identical between years), we treated these years as two experiments rather than two treatments of an experimental factor.

We calculated the absolute growth rate as the slope of length vs. experimental day (mm/day). We estimated it based on experimental day 6 for 2018 and days 9 to 18 and 19 for 2019. We chose these growth periods to maximize sample size and capture the rapid growth phase of larvae. The sample size in 2018 decreased drastically after day 6 due to mortality and/or wandering off the plant. In 2019, we decided to delay the first measurement until day 9 of the experiment to reduce mortality of the early instars and we were not able to check larvae in all treatment levels on the same final day, so we had to use different final experimental days for the big and medium (day 18) treatment levels vs. for the small (day 19) treatment level.

Where data did not meet model assumptions, generalized linear models were used, otherwise linear models were used. To model differences in larval development time and the absolute growth rate as a function of milkweed size, we used a gamma error distribution, appropriate for continuous, positive values. For survival, we used a binomial error distribution. To determine potential differences in leaf quality amongst plants of different sizes, we modeled latex exudation and leaf thickness as a function of average milkweed height for each treatment level using linear models. To compare differences between levels of categorical variables, we used a TukeyHSD pairwise comparison test.

## 3 RESULTS

### 3.1 Observational study

We surveyed 2851 milkweed plants across all sites and monarch eggs were present at 75% of the sites (18/24; *n* = 62 eggs; median = 2.5 [2.6 SD] eggs per location). We also observed 51 larvae and 72 adults. We observed monarch eggs throughout the entire sampling period. The seasonal distribution of monarch egg observations was best predicted by a unimodal, quadratic relationship (ΔAICc = 37.1, Table S2; Figure 2a). They peaked in the third sampling period (66% of total observations occurred during July 15–July 26; *n* = 41; Figure 2a; Figure S2).

The milkweed variables that predicted monarch occurrence were: (1) developmental stage; (2) height; and (3) leaf discolouration (Table 1; Figure 2). Egg and larval presence was highest on plants in earlier developmental stages (prebud, bud; χ2‐test_[3]_ = 20.4, *p* < .0001; Table 1, Figure 2b). Egg presence was highest on shorter plants (β = −1.61 (0.54SE); χ2‐test _[1]_ = 8.82, *p* = .003), and plants with lower levels of leaf discolouration (0, <5%; χ2‐test_[4]_ = 5.71, *p* = .01) (Table 1, Figure 2). The number of egg observed peaked (Figure 2a; Figure S2) when 57% of plants were in early developmental stages (prebud, bud), the mean height of plants was 57 cm ± 1.7 SE, and 48% of plants had low discolouration (0, <5%) (Figure 3). Percentage of herbivory, leaf number and number of plants did not predict monarch occurrence (Table 1).

The seasonal pattern of progression through stages differed across milkweed characteristics. The proportion of plants in later developmental stages (i.e., anthesis, postanthesis) increased through the season (Figure 3a). However, the majority of plants surveyed over the season were in early developmental stages (mean: 52%; prebud, bud). Even in the last sampling period, 52% of plants were prebud. Plants peaked in height mid‐season (Table S1; Figure 3b); however, shorter plants (as defined by the mean size of prebud plants: 48 cm [23SD]) were available throughout the season (Figure 3b). The availability of plants with low levels of discolouration (0, <5%) decreased through the season (Figure 3c), ranging from 88% to 1% (mean: 22%) over the season.

### 3.2 Field experiments

**FIGURE 4:**
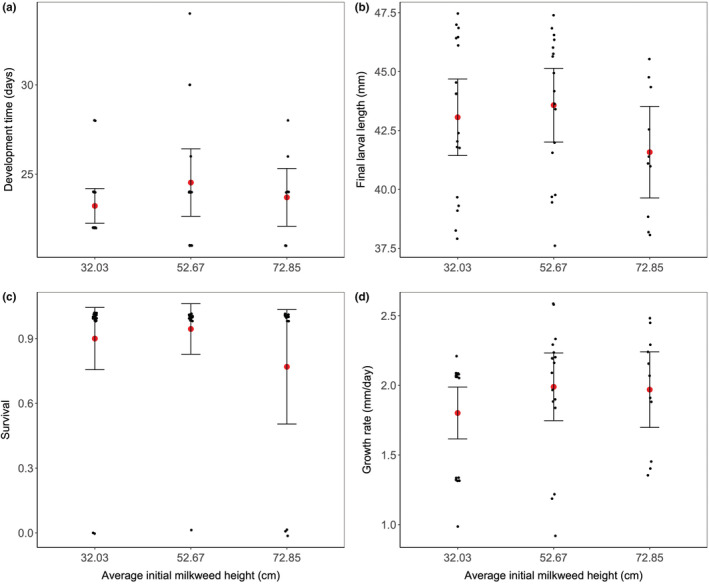
Differences in larval performance based on milkweed size from the 2019 experiments. Shown is larval performance estimated as: (a) development time (days), (b) final larval length (mm), (c) survival, and (d) absolute growth rate (mm/days). AGR was calculated over a shorter time period than development time or final larval length. Milkweed size is represented by size treatment levels (small = 32.03 cm; medium = 52.67 cm; big = 72.85 cm). Milkweed height is represented as mean initial height per treatment level. Shown are predicted mean values (red dots) with 95% confidence intervals and individual data points (black dots, jittered).

Milkweed height did not have an effect on any of the larval performance estimates in either year (Table 2; Figure 4).

**TABLE 2:** Results from generalized linear and linear models predicting monarch performance based on milkweed size. Monarch performance was estimated as development time (days), final larval length (mm), absolute growth rate (mm/days), and survival for both 2018 and 2019 experiments. Milkweed size is represented by size treatment levels. Generalized linear models were used for larval development time (gamma error distribution), absolute growth rate (gamma error distribution), and survival (binomial error distribution), and linear models were used for final larval length

Year	Response variable	Sample size (*n*)				Test statistic	*p* value (df)
		Small treatment level	Medium treatment level	Big treatment level	Total sample size		
2018	Development time (days)	8	5	N/A	13	χ ^2^ = 0.01	.93 (1)
	Final larval length (mm)	8	5	N/A	13	F_1,11_ = 0.06	.82
	Absolute growth rate (mm/day)	8	8	N/A	16	χ ^2^ = 2.09	.15 (1)
	Survival	8	5	N/A	13	χ ^2^ = 1.72	.19 (1)
2019	Development time (days)	18	17	10	45	χ ^2^ = 0.45	.5 (1)
	Final larval length (mm)	18	17	10	45	F_2,43_ = 0.97	.33
	Absolute growth rate (mm/day)	20	17	11	48	χ ^2^ = 1.46	.23 (1)
	Survival	18	17	10	45	χ ^2^ = 0.98	.32 (1)

**FIGURE 5:**
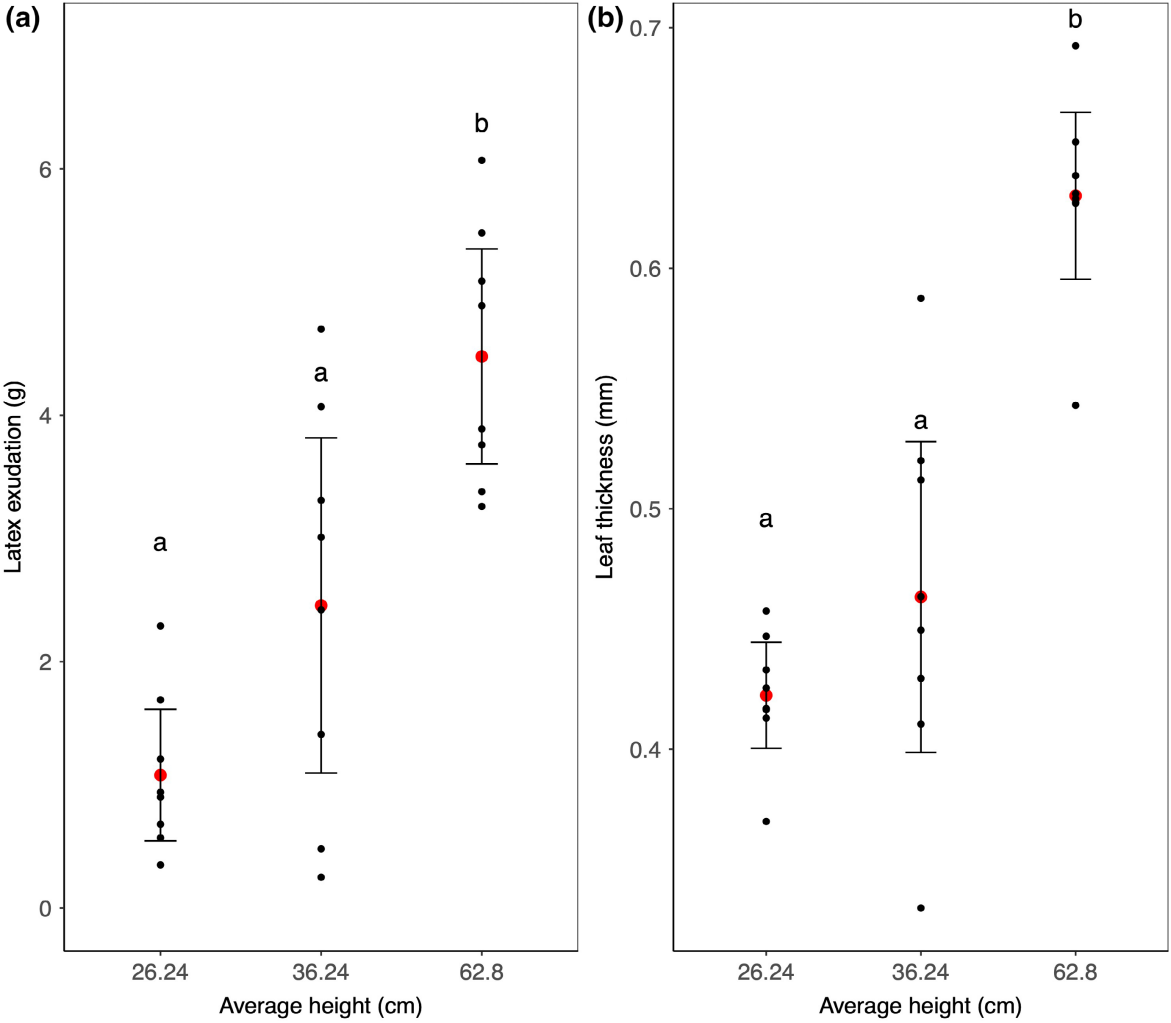
The effect of common milkweed plant size on leaf quality (*n* = 8 plants per treatment level). Leaf quality is measured as (a) latex exudation (g) and (b) leaf thickness (mm) (b). Milkweed size is represented as mean height (cm) per treatment level. Heights were measured on September 13, 2019. Shown are mean values (red dots) with 95% confidence intervals and individual data points (black dots). Letters represent significant pairwise comparisons (*p* < .05).

Latex exudation (*F*_2,21_ = 16.91, *p* < .0001) and leaf thickness (*F*_2,21_ = 34.6, *p* < .0001) differed across milkweed size treatment. There was 76% more latex exuded from leaves on big plants than small (3.78 mg, *t*_21,2_ = 5.78, *p* < .0001) and 45% more than medium plants (1.37 mg, *t*_21,2_ = 3.44, *p* = .007) (Figure 5a). Big plants also had 33% thicker leaves than small (0.235 mm, t_21,2_ = 7.85, *p* < .0001) and 26% thicker leaves than medium plants (0.105 mm, *t*_21,2_ = 6.31, *p* < .0001) (Figure 5b).

## 4 DISCUSSION

The potential for fitness consequences for the monarch due to phenological asynchrony with milkweed as a result of climate change has not been well studied. Our study contributes two main findings that suggest the eastern population of monarchs may not face negative consequences if asynchrony with milkweed were to occur. First, we found that the preferred plant types for oviposition are available through the breeding season. Second, we found that despite an increase in plant defenses with plant size, larval performance was not reduced on larger plants.

### 4.1 Oviposition preference

We found more eggs on shorter milkweed plants in earlier developmental stages (i.e., prebud, bud), and with less discolouration (i.e., fewer yellow leaves). This suggests that females prefer ovipositing on these plant types, though a choice experiment is needed to determine this with certainty. Our results are consistent with other studies that found monarchs prefer to oviposit on milkweed plants in earlier stages of development (e.g., Bergström et al., 1994; Dixon et al., 1978; Zalucki & Kitching, 1982) and on shorter plants (e.g., Zalucki & Kitching, 1982; Knight et al., 2019 but see Cohen & Brower, 1982; Malcolm & Brower, 1986). Bergström et al. (1994) linked this preference to the attractiveness of the volatile compounds emitted from young milkweed plant leaves compared with old milkweed plant leaves. While monarch larvae have evolved various mechanisms that allow them to overcome and sequester some of the milkweed's defensive compounds (Brower et al., 1967; Dobler et al., 2012; Zalucki & Brower, 1992), the survival rate of first instars is between 3 to 40%. This low survival rate is likely due to other plant defensive traits such as trichomes (Rathcke & Poole, 1975) and leaf toughness (Clissold et al., 2009). Monarchs may be attracted to younger plants because as milkweed develops, their defensive traits increase while their nutritive content decrease, thus causing a decline in their overall quality (Figure 5; Yang et al., 2020). Ultimately, the ingestion of milkweed poses a trade‐off between toxicity and protection against natural enemies (Despland, 2017). Alternatively, ovipositing monarchs may select plants that reduce predation risk for their offspring rather than selecting them for their food quality (e.g., Haan & Landis, 2019). Monarch predators (e.g., ants, arachnids, beetles, and true bugs) have been found to be less abundant on younger milkweed plants relative to older plants (Haan & Landis, 2019).

Consistent with other studies (e.g., Fischer et al., 2015), we found leaf discolouration to be an important factor in determining oviposition preference. The degree of leaf discolouration typically indicates how close a plant is to senescence. Senescence is associated with physiological changes such as the redistribution of nutrients (e.g., nitrogen) (Guiboileau et al., 2010; Hill, 1980). Leaf discolouration can also indicate disease (Häffner et al., 2015), herbivory (Agrawal & Van Zandt, 2003), nutrition deficiency (Noodén et al., 1997), or competition (which also causes nutrient deficiency) (Noodén et al., 1997). These factors can all decrease plant quality for herbivores (Mattson, 1980).

Despite leaf number being correlated with plant height (Table S1), we did not find an effect of leaf number on the occurrence of eggs on milkweed plants. To our knowledge, no other study has considered the effect of milkweed leaf number on oviposition preference. Quantity of food is important for young instars that are often incapable of traveling to a new host until they are older (Fisher et al., 2020; Futuyma et al., 1984). However, more functional measures of plant size, such as total leaf area, may be a better predictor of monarch oviposition preference on common milkweed (Cohen & Brower, 1982; Yang et al., 2020). Plants with larger leaves may provide larvae with greater protection from direct sunlight, thus reducing the potential risk of desiccation (Cohen & Brower, 1982). In any case, our results suggest that number of leaves is not the best predictor of oviposition preference on common milkweed in this area.

We did not find a relationship between oviposition preference and herbivore damage. This is inconsistent with previous studies, which have found fewer monarch eggs on herbivore‐damaged plants (He & Agrawal, 2020). However, He and Agrawal (2020) did not quantify the degree of damage from herbivory in their experiment. Therefore, it could be that the levels of herbivory we observed were not high enough on the plants at the time of oviposition to deter females. Alternatively, quantifying herbivore damage in the field is prone to a measurement error, which could have affected our ability to detect a relationship (Zvereva & Kozlov, 2019). As plants experience increasing damage from herbivory, their quality as a food source for herbivorous larvae decreases (Karban & Baldwin, 1997). Common milkweed has been shown to increase latex production in response to herbivory damage, which can negatively impact monarch larval growth (Van Zandt & Agrawal, 2004).

### 4.2 Seasonal availability of preferred milkweed plants

Plants with preferred characteristics for oviposition were generally available, and in large proportion, throughout the season. This result suggests that even if the relative timing of the monarch–milkweed interaction in the eastern population shifts due to climate change, there may be suitable milkweed plants available for oviposition throughout the breeding season in this region, thus reducing potential consequences for the monarch. We note two caveats here. First, our surveys missed the first part of the milkweed growing season so the availability of preferred plants if the arrival of the first migrants is earlier than normal is unclear, though plants in earlier developmental stages are unlikely to be in short supply. Second, as we did not examine whether preference changes over the season, it is unclear whether the preferred plants we identified here have the same attractiveness at the end of the season. Nonetheless, monarchs have been consistently shown to prefer to oviposit on younger or regenerating stems so there is unlikely to be a seasonal effect (e.g., Bergström et al., 1994; Haan & Landis, 2019).

Anecdotal observations of milkweed communities in Northern California suggest that those plants are more seasonally synchronized than in this region (e.g., young plants are only available early in the season; L. Yang, pers. comm.). Thus, further study of seasonal patterns in milkweed development in other areas of the monarch's range is needed, especially given that milkweed populations in Canada (Lalonde et al., 2021) and the United States (Pleasants & Oberhauser, 2013; Zaya et al., 2017) have declined in recent decades. However, given that our study was done at the northern range limit of the monarch (Figure 1), where the growing season is the shortest, evaluating seasonal availability further south may not be required.

### 4.3 Larval performance

We found that larger plants exuded more latex and had thicker leaves than smaller plants. This is consistent with other studies that have found that defensive traits in milkweed increase over the ontogeny of the plant (e.g., Yang & Cenzer, 2020; Zalucki & Brower, 1992). However, the higher expression of these defensive traits in larger plants did not translate into reduced larval performance in larger plants. This is inconsistent with previous work, which found higher larval performance in younger milkweed plants (Dixon et al., 1978; Yang et al., 2020; Yang & Cenzer, 2020), plants with lower latex and/or cardenolide concentrations (e.g., Malcolm & Zalucki, 1996; Zalucki & Brower, 1992) and thinner leaves (Agrawal & Fishbein, 2006).

Our results may be inconsistent with previous experiments because of differences in the type and degree of manipulation used across experiments, and the presence of compensatory feeding. First, as our experiments in each year did not have true replication, the lack of treatment effect on larval performance may not apply to other local sites if they have a different history or soil type. Second, previous studies directly tested the effect of plant age on performance by propagating their plants from seed (Yang et al., 2020; Yang & Cenzer, 2020), whereas we were not able to control for plant age with mowing. It could be that there are multi‐generational effects on plant quality in this milkweed species such that plant age and size are not well correlated (e.g., small, old plants). Third, previous studies used larger differences between plant treatment levels than we did (e.g., > 3 weeks: Yang et al., 2020; Yang & Cenzer, 2020). Therefore, it is possible that the plant quality differences between our treatment levels were not great enough to affect larval performance. Fourth, larvae may have compensated for lower leaf quality in the large size treatment level by consuming more leaves (i.e., compensatory feeding; Slansky & Wheeler, 1991). The consumption of more leaves in the large plant size treatment could have increased the performance of larvae in that treatment resulting in no difference in performance between treatment levels. This strategy has been previously demonstrated in monarch larvae reared on common milkweed plants with lower nitrogen levels (Lavoie & Oberhauser, 2004).

Finally, there were a high number of egg hatch failures, particularly in 2018 (average egg hatch failure: 26.6% in 2018, 22.5% in 2019), which decreased the total sample size (Table 2) and the precision of our estimates of performance. However, given the low abundance of monarchs in the region, there were constraints on the number of monarch eggs available in a short period of time.

Overall, we did not find support for the preference‐performance hypothesis (i.e., females prefer to oviposit on plants, which will allow their offspring to achieve the highest performance; Levins & MacArthur, 1969). Our results from the observational study suggest that females prefer to oviposit on shorter milkweed plants; yet, we did not find higher performance on shorter plants in the experiment. These results are consistent with Jones and Agrawal (2019), which tested the hypothesis across plant species. However, both of our studies controlled for predation. Future studies should explore the relative importance of predation on larval performance, and measure oviposition preference and larval performance in the same plants.

In conclusion, while it is unclear how the relative timing of the milkweed–monarch interaction will change in the future, our results suggest that shifts in phenological synchrony within the breeding season may not have negative consequences on larval performance. Future experiments should increase replication and determine whether performance may be more impacted later in the season on older, unmanipulated stems, and on older plants throughout the season to replicate the scenario where milkweed spring phenology advances to a greater degree than the arrival of monarchs. Additionally, future studies should expand this work to consider the seasonal availability of preferred plants across an urbanization gradient given the impact of urbanization on plant phenology (Li et al., 2019) and to better guide conservation strategies. Finally, it remains unclear how climate change will affect the seasonal availability and quality of milkweed plants (Kharouba & Yang, 2021).

## AUTHOR CONTRIBUTIONS

**Sydney M. Gilmour:** Conceptualization (equal); data curation (lead); formal analysis (lead); funding acquisition (supporting); investigation (equal); methodology (equal); project administration (supporting); resources (supporting); software (lead); supervision (supporting); validation (lead); visualization (lead); writing – original draft (lead); writing – review and editing (supporting). **Heather M. Kharouba:** Conceptualization (equal); data curation (supporting); formal analysis (supporting); funding acquisition (lead); investigation (equal); methodology (equal); project administration (lead); resources (lead); software (supporting); supervision (lead); validation (supporting); visualization (supporting); writing – original draft (supporting); writing – review and editing (lead).

## Supporting information


Table S1
**Table S2**.
**Table S3**.
Figure S1.
Figure S2.

## Data Availability

Data supporting the results are archived in Dryad, accessible at: doi:10.5061/dryad.02v6wwq61.
