# Supplementary material for: Eastern monarch larval performance may not be affected by shifts in phenological synchrony with milkweed
Source: Ecol Evol. 2022 Aug 4;12(8):e9131. doi: 10.1002/ece3.9131 (PMC9351326; doi:10.1002/ece3.9131)

# Appendix

**Table S1**. Test of non-independence between milkweed characteristic predictor variables. Relationship between milkweed-specific variables and day of year (linear and its quadratic term), also shown. Each row represents an individual model. All models included day of year (linear and quadratic term) as a fixed effect and site as a random effect. Test statistics and model types are also shown. In models with leaf number as a response, it was square root transformed to improve normality.

| **Variable 1 (response)** | **Variable 2 (predictor)** | **Test statistic** | **P value** | **Variable types** | **Model** |  |
| --- | --- | --- | --- | --- | --- | --- |
| Height (cm) | Day of year +  Day of year^2^ | LRT=198.8 | <0.0001 | Continuous vs. continuous | Linear mixed model |  |
|  | Leaf number | F_1,2824_=1169.25 | <0.0001 | Continuous vs. continuous | Linear mixed model |  |
|  | Developmental stage | F_3,2822_=738.39 | <0.0001 | Continuous vs. categorical | Linear mixed model |  |
|  | Herbivory | F_3,2822_=15.2 | <0.0001 | Continuous vs. categorical | Linear mixed model |  |
|  | Leaf discolouration | F_4,2821_=14.63 | <0.0001 | Continuous vs. categorical | Linear mixed model |  |
| Leaf number | Day of year +  Day of year^2^ | LRT=525.41 | <0.0001 | Continuous vs. continuous | Linear mixed model |  |
|  | Developmental stage | F_3,2822_=85.95 | <0.0001 | Continuous vs. categorical | Linear mixed model |  |
|  | Herbivory | F_3,2822_=15.52 | <0.0001 | Continuous vs. categorical | Linear mixed model |  |
|  | Leaf discolouration | F_4,2821_=16.89 | <0.0001 | Continuous vs. categorical | Linear mixed model |  |
| Developmental stage | Day of year + Day of year^2^ | LRT=55.94 | <0.0001 | Categorical vs. continuous | Ordinal logistic mixed regression |  |
|  | Herbivory | $\chi$^2^ =17.54 | <0.001 | Categorical vs. categorical | Ordinal logistic mixed regression |  |
|  | Leaf discolouration | $\chi$^2^=40.7 | <0.0001 | Categorical vs. categorical | Ordinal logistic mixed regression |  |
| Herbivory | Day of year + Day of year^2^ | LRT=6 | 0.01 | Categorical vs. continuous | Ordinal logistic mixed regression |  |
|  | Leaf discolouration | $\chi$^2^=5.87 | 0.209 | Categorical vs. categorical | Ordinal logistic mixed regression |  |
| Leaf discolouration | Day of year + Day of year^2^ | LRT=3.15 | 0.07 | Categorical vs. continuous | Ordinal logistic mixed regression |  |

**Table S2**. The results of model selection to predict monarch egg occurrence (presence and absence) on milkweed plants (*n*=24 sites) based on day of year. Models were selected between a reference model and a more complex model based on ΔAICc.

| Reference model | Model | Estimate (SE) | AICc | ΔAICc | χ2-test | P value (df) | R^2^ |
| --- | --- | --- | --- | --- | --- | --- | --- |
| Intercept-only |  | N/A | 588.8 | 0 |  |  |  |
|  | + Day of year | -1.63 (0.56) | 582.05 | 6.76 | 8.76 | 0.0031 | 0.20 |
| Day of year |  | N/A | 582.05 | 0 |  |  |  |
|  | + Day of year^2^ | Linear: 89.7 (14.7) | 545.0 | 37.05 | 8.8* | 0.003 (1)* | 0.20 |
|  |  | Quadratic: -22.7 (3.7) |  |  | 39.1** | <0.0001 (1)** | 0.52 |

**Table S3**. Post-hoc Tukey pairwise comparison results associated with Figure 2, the relationship between egg occurrence and milkweed plant characteristics. Estimates are on the logit scale and not response scale. Significant comparisons (p<0.05) are in bold.

| Predictor variable | Comparisons between levels | Estimate (SE) | Z ratio | P value |
| --- | --- | --- | --- | --- |
| Leaf discolouration (%) | 0 and <5 | -0.3 (0.38) | -0.78 | 0.94 |
|  | 0 and 5-40 | 0.42 (0.45) | 0.93 | 0.88 |
|  | 0 and 40-80 | 1.49 (0.7) | 2.12 | 0.21 |
|  | 0 and 80-100 | 1.21 (1.1) | 1.1 | 0.81 |
|  | <5 and 5-40 | 0.72 (0.35) | 2.08 | 0.23 |
|  | **<5 and 40-80** | **1.79 (0.63)** | **2.84** | **0.04** |
|  | <5 and 80-100 | 1.51 (1.05) | 1.44 | 0.6 |
|  | 5-40 and 80-100 | 1.07 (0.65) | 1.65 | 0.47 |
|  | 5-40 and 80-100 | 0.78 (1.06) | 0.74 | 0.95 |
|  | 40-80 and 80-100 | -0.28 (1.17) | -0.24 | 1.0 |
| Developmental stage | Prebud and bud | -0.25 (0.27) | -0.94 | 0.78 |
|  | **Prebud and anthesis** | **1.31 (0.48)** | **2.76** | **0.03** |
|  | **Prebud and post-anthesis** | **1.18 (0.44)** | **2.66** | **0.039** |
|  | **Bud and anthesis** | **1.57 (0.50)** | **3.11** | **0.01** |
|  | **Bud and post-anthesis** | **1.44 (0.49)** | **2.93** | **0.018** |
|  | Anthesis and post-anthesis | -0.13 (0.63) | -0.21 | 0.99 |

**Figures**

**Figure S1.** The height distribution of common milkweed from observational surveys in 2019 and initial heights from the field experiment in early August 2018 and 2019. Shown in grey is the height distribution (cm) from milkweed (*n*=114 plants) measured at seven sites around Ottawa on August 11^th^, 2019. Red, blue and black vertical lines represent the mean initial heights at the start of experiments from the small, medium and big plant treatment levels, respectively. Solid lines are mean initial heights at the start of the experiment on July 30^th^, 2018 (small plants (red) 39 cm $\pm$ 1.33 SE, medium plants (blue): 59 cm $\pm$ 0.75 SE). Dashed lines are mean initial heights at the start of the experiment on August 10^th^, 2019 (small treatment plants (red): 32.0 cm $\pm$ 2.8 SE, medium treatment plants (blue): 52.7 cm $\pm$ 3.5 SE, big treatment plants (black): 72.9 cm $\pm$ 4.4 SE).

**Figure S2.** The number of monarch eggs observed (*n*=62) across the season. Shown are monarch egg counts for every 12-to-14-day sample period.


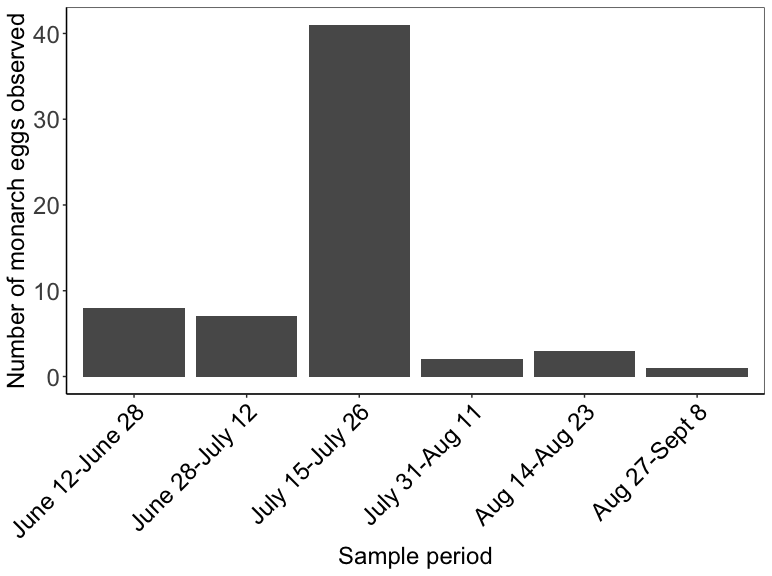

Supplement: Supplementary file 1 — Table S1 Table S2. Table S3. Figure S1. Figure S2. [file ECE3-12-e9131-s001.docx]
